# Expression of N471D strumpellin leads to defects in the endolysosomal system

**DOI:** 10.1242/dmm.033449

**Published:** 2018-09-13

**Authors:** Lin Song, Ramesh Rijal, Malte Karow, Maria Stumpf, Oliver Hahn, Laura Park, Robert Insall, Rolf Schröder, Andreas Hofmann, Christoph S. Clemen, Ludwig Eichinger

**Affiliations:** 1 Center for Biochemistry, Institute of Biochemistry I, Medical Faculty, University of Cologne, 50931 Cologne, Germany; 2 Department of Biology, Texas A&M University, College Station, TX 3258, USA; 3 Max Planck Institute for Biology of Ageing, Biological Mechanisms of Ageing, 50931 Cologne, Germany; 4 CR-UK Beatson Institute, Institute of Cancer Sciences, Glasgow University, Glasgow G12 8QQ, UK; 5 Institute of Neuropathology, University Hospital Erlangen, 91054 Erlangen, Germany; 6 Structural Chemistry Program, Eskitis Institute, Griffith University, N75 Don Young Road, Nathan, QLD 4111, Australia; 7 Faculty of Veterinary Science, The University of Melbourne, Parkville, VIC 3030, Australia; 8 Department of Neurology, Heimer Institute for Muscle Research, University Hospital Bergmannsheil, Ruhr-University Bochum, 44789 Bochum, Germany

**Keywords:** Spastic paraplegia 8, Strumpellin, WASH complex, *Dictyostelium discoideum*, Endolysosomal system, Exocytosis, Lysosome

## Abstract

Hereditary spastic paraplegias (HSPs) are genetically diverse and clinically characterised by lower limb weakness and spasticity. The N471D and several other point mutations of human strumpellin (Str; also known as WASHC5), a member of the Wiskott–Aldrich syndrome protein and SCAR homologue (WASH) complex, have been shown to cause a form of HSP known as spastic paraplegia 8 (SPG8). To investigate the molecular functions of wild-type (WT) and N417D Str, we generated *Dictyostelium* Str^−^ cells and ectopically expressed Str^WT^-GFP or Str^N471D^-GFP in Str^−^ and WT cells. Overexpression of both proteins apparently caused a defect in cell division, as we observed a clear increase in multinucleate cells. Real-time PCR analyses revealed no transcriptional changes in WASH complex subunits in Str^−^ cells, but western blots showed a twofold decrease in the SWIP subunit. GFP-trap experiments in conjunction with mass-spectrometric analysis revealed many previously known, as well as new, Str-interacting proteins, and also proteins that no longer bind to Str^N471D^. At the cellular level, Str^−^ cells displayed defects in cell growth, phagocytosis, macropinocytosis, exocytosis and lysosomal function. Expression of Str^WT^-GFP in Str^−^ cells rescued all observed defects. In contrast, expression of Str^N471D^-GFP could not rescue lysosome morphology and exocytosis of indigestible material. Our results underscore a key role for the WASH complex and its core subunit, Str, in the endolysosomal system, and highlight the fundamental importance of the Str N471 residue for maintaining lysosome morphology and dynamics. Our data indicate that the SPG8-causing N471D mutation leads to a partial loss of Str function in the endolysosomal system.

This article has an associated First Person interview with the first author of the paper.

## Abstract (precis)

**Summary:** Strumpellin, a core member of the WASH complex, plays a key role in the endolysosomal system. Deficiencies in endo-/exocytosis and lysosome morphology are caused by the N471D strumpellin mutation, which is associated with spastic paraplegia 8.

## INTRODUCTION

The use of model organisms for the molecular analysis of disease-causing mutations in human genes has strongly increased in recent years. Generally, these mutations can only be studied in a very limited way in patients, and even in mouse models their analysis is expensive, time consuming and technically challenging, or sometimes even impossible. In contrast, their functional analysis in simple model organisms is often easier, faster and cheaper (Bonini and Gitler, 2011). *Dictyostelium* amoebae grow as separate, independent cells and take up bacteria via phagocytosis (Kessin, 1981). Upon starvation, *Dictyostelium* *discoideum* cells aggregate and undergo a series of defined morphological states, finally giving rise to a mature fruiting body which is composed of several distinct cell types (Annesley and Fisher, 2009). Despite its lower complexity, the organism is similar to higher eukaryotes in many cellular aspects and is, therefore, increasingly used to analyse the molecular consequences of disease-causing mutations in human genes (Mesquita et al., 2017; Müller-Taubenberger et al., 2013; Williams et al., 2006).

**Fig. 1.**
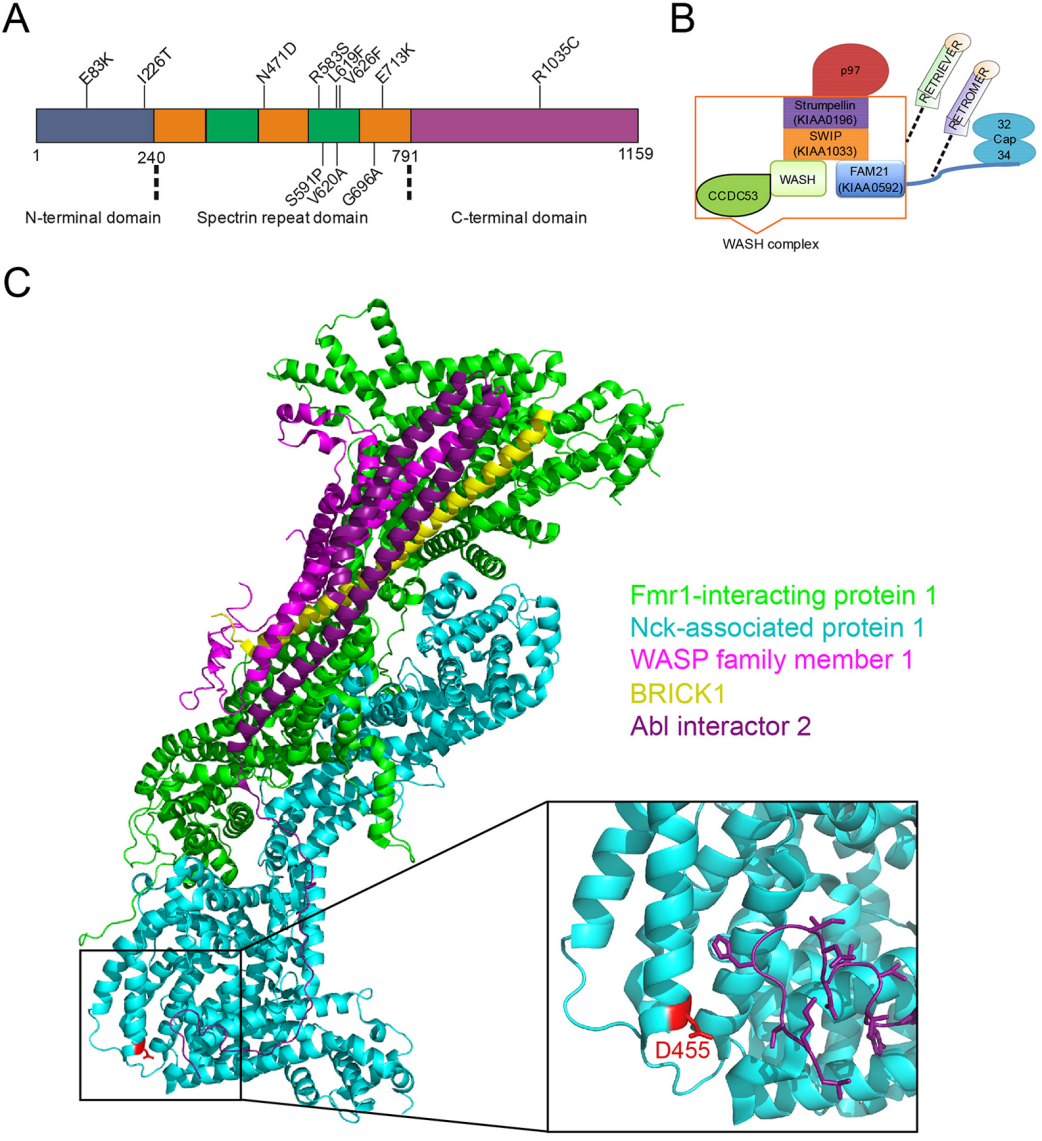
**Schemes of strumpellin and the WASH complex, and three-dimensional structure of the WAVE complex.** (A) Domain structure of strumpellin. Strumpellin is composed of an N-terminal domain, a middle domain with five spectrin-like repeats and a C-terminal domain. The amino acids of the SPG8-associated point mutations are shown at their approximate positions. (B) Model of the WASH complex and its associated complexes and proteins. The five core proteins – WASH, FAM21 (KIAA0592), CCDC53, SWIP (KIAA1033) and strumpellin (KIAA0196) – of the WASH complex, as well as two of the known interacting proteins, Cap32/34 and p97, are depicted. For completeness, the retromer and retriever complexes, which very likely interact with the WASH complex in a mutually exclusive manner, are also shown. (C) Representation of the WAVE complex (PDB 3p8c), showing the different components of the complex in different colours. The hetero-pentameric WAVE regulatory complex is composed of Fmr1-interacting protein 1 (also known as Sra1, green), Nck-associated protein 1 (Nap1, turquoise), WASP family member 1 [WASF1 (also known as WAVE1), pink], Abl interactor 2 (Abl2, purple) and HSPC300 (BRICK1, yellow). These proteins correspond to SWIP, strumpellin, WASH, FAM21 and CCDC53, respectively, in the WASH complex. Nap1 D455 is depicted in red. Figure prepared with PyMOL (DeLano, 2002) and Inkscape (https://inkscape.org/en/).

Hereditary spastic paraplegias (HSPs) are a large group of hereditary genetic disorders and can be inherited in an autosomal-dominant, autosomal-recessive or X-linked-recessive manner. Over 70 genotypes have been described, and over 50 genetic loci have been linked to HSPs (Lo Giudice et al., 2014). The diseases are caused by upper motor neuron degeneration and characterised by progressive spasticity of the lower limbs. Based on additional neurological features, HSPs are classified into pure and complicated forms (de Souza et al., 2017). They can be further categorised based on symptoms, age of onset, affected genes and biochemical pathways involved (Blackstone, 2012). Spastic paraplegia 8 (SPG8, OMIM #603563) is an autosomal dominant form of HSP and is caused by mutations in *KIAA0196* (also known as strumpellin or *WASHC5*) (Valdmanis et al., 2007)*.* The gene is located on chromosome 8q24.13 and encodes strumpellin, an evolutionarily conserved 1159-amino acid protein with a calculated molecular mass of 134 kDa. Based on predicted secondary structure elements, strumpellin can be divided into three parts: an N-terminal part, from amino acid 1 to 240; a central part, from residue 241 to 791, with five spectrin-like repeats; and a C-terminal part. Until now, 11 strumpellin point mutations and one exonic deletion have been identified in a total of 16 families, of which most cause a pure motor form of HSP (Bogucki and Sobczyńska-Tomaszewska, 2017; Jahic et al., 2014; Valdmanis et al., 2007). An additional strumpellin splice site mutation has been identified as the cause of a form of the Ritscher–Schinzel syndrome (Elliott et al., 2013). Of the SPG8-causing point mutations, only the V626F and V620A mutations occur in four and two families, respectively; all other mutations have each been identified in only one family. Eight of the point mutations, including the N471D mutation, are localised in the spectrin-like repeats (Fig. 1A).

Strumpellin is part of a large five-protein assembly, the Wiskott–Aldrich syndrome protein and SCAR homologue (WASH) complex, the core members of which are WASH (also known as WASHC1), FAM21 (WASHC2, KIAA0592), coiled coil domain containing 53 (CCDC53; WASHC3), strumpellin and WASH-interacting protein (SWIP; WASHC4, KIAA1033), and strumpellin (WASHC5, KIAA0196) (Derivery et al., 2009; Gomez and Billadeau, 2009). Furthermore, the hexameric triple-A ATPase p97 (or valosin-containing protein, VCP; also known as TER94 *in Drosophila melanogaster* and CDC48 in *Saccharomyces cerevisiae*) directly interacts with strumpellin and the F-actin capping protein heterodimer Cap32/34 with FAM21 (Clemen et al., 2010; Derivery et al., 2009). Recently, it has also been shown that the WASH complex interacts with the retromer and retriever complexes (Lee et al., 2016; McNally et al., 2017) (Fig. 1B). The WASH complex is highly conserved from amoeba to man (Derivery and Gautreau, 2010; Insall, 2013). It plays essential roles in the endosomal system, such as the recycling of the vacuolar ATPase (v-ATPase) from postlysosomes prior to exocytosis or of internalised receptor molecules back to the cell surface (Carnell et al., 2011; Derivery et al., 2012, 2009; Rotty et al., 2013). Sorting of the v-ATPase and other cargo molecules appears to be accomplished through WASH complex-mediated F-actin polymerisation (Carnell et al., 2011; Gomez and Billadeau, 2009). The complex is also required for the structural integrity of endosomes and lysosomes (Duleh and Welch, 2010; Gomez et al., 2012). Disruption of either one of the five *D. discoideum* WASH complex members revealed a complete block in exocytosis for each of the generated knockout mutants (Carnell et al., 2011; Park et al., 2013).

We have chosen to study the strumpellin N471D mutation in the model organism *D. discoideum* because the region around the N471 residue is highly conserved across species (Fig. S1). A further reason was that we have generated, in a parallel project, knock-in mouse models for this mutation and are currently in the process of analysing the mutant mice. Here, we analysed the *D. discoideum* Str^−^ mutant as well as strains that express Str^WT^-GFP or Str^N471D^-GFP in AX2 wild-type (WT) and Str^−^ cells. We find severe endolysosomal defects in Str^−^ cells and in Str^−^ cells expressing Str^N471D^-GFP, which include a block in exocytosis, and changes in v-ATPase distribution, lysosome morphology and secretion of lysosomal enzymes. We conclude that the Str^N471^ residue plays a crucial role in some of the WASH complex-mediated dynamic transitions in the endolysosomal system.

## RESULTS

### Structural appraisal

In the absence of three-dimensional structural information for strumpellin, we have previously conducted a structural appraisal of this protein and found an overall topology consisting of three different folds: an N-terminal part, from amino acid 1 to 240, five spectrin-like repeats in the central region (residues 241-791) and a C-terminal part (Fig. 1A) (Clemen et al., 2010). Given the large size of this protein, the absence of any particular features that might serve as structural anchor points and the lack of reasonably identical three-dimensional protein structures, generation of a ‘homology model’ of strumpellin does not appear feasible at this time.

Notwithstanding the challenge of generating an atomic model by comparative modelling, it is possible to identify three-dimensional protein structures that illustrate the overall fold of the strumpellin spectrin-like domain. Using pGenTHREADER (Lobley et al., 2009), we identified the Nck-associated protein 1 (Nap1; also known as NCKAP1, membrane-associated protein HEM-2, p125Nap1; UniProt Q9Y2A7) as a structural homologue of the strumpellin spectrin-like domain (Fig. S2). Nap1 adopts a spectrin fold and is part of the WAVE complex that regulates lamellipodia formation. By inference, a likely role of N471 of strumpellin can be postulated based on the three-dimensional crystal structure of the actin regulatory WAVE complex (Fig. 1C) (Chen et al., 2010). The WAVE complex is homologous to the WASH complex and the five core members of both complexes share sequence similarity.

The structure-based amino acid sequence alignment of Nap1 and strumpellin shows that residue D455 of Nap1 corresponds to the strumpellin residue N471 (inset in Fig. 1C; asterisk in Fig. S2). In the WAVE complex, the Nap1 D455 residue is at the start of an α-helix and forms part of the interaction interface with Abl interactor 2. One can thus infer that N471 might be involved directly in specific protein-protein interactions of strumpellin in the WASH complex, possibly with Fam21. The expectation that the exposure of a negative charge by strumpellin (D471) would weaken or disrupt such interaction was, however, not confirmed by our results from immunoprecipitation experiments (see below).

### SWIP levels are decreased in strumpellin knockout cells

**Fig. 2.**
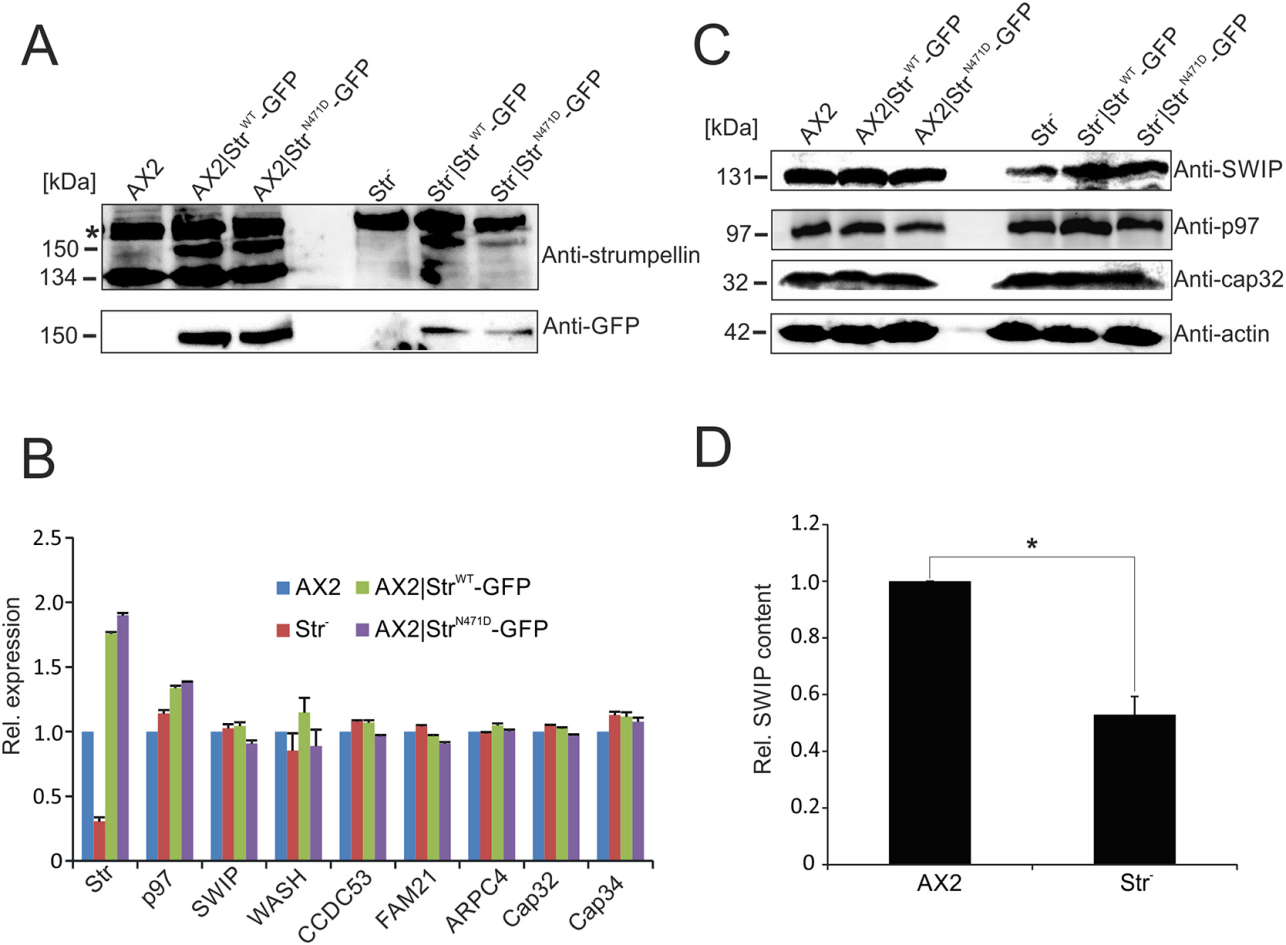
**Immuno-verification and real-time PCR analysis of mutant *Dictyostelium* strains.** (A) Verification of generated *D. discoideum* strains by western blot analysis with anti-strumpellin and anti-GFP antibodies. The asterisk indicates an unspecific band. (B) Real-time PCR analysis of AX2 WT and mutant strains. The relative mRNA expression levels of the genes encoding strumpellin, p97, members of the WASH complex, ARPC4 and Cap32/34 in *D. discoideum* cells are depicted. The expression level in AX2 was set to 1. Means±s.d. of three independent determinations are depicted. (C) Immunoblot analyses of SWIP, p97 and Cap32 in the generated *D. discoideum* strains using specific antibodies. Actin (lower panel) served as a loading control. (D) Quantitation of SWIP expression in AX2 WT and Str^−^ cells. Means±s.e.m. of three independent experiments are depicted. Significance was calculated using Student’s *t*-test. *P≤0.05.

To analyse the cellular function of strumpellin, we generated strumpellin knockout cells (Str^−^) as well as strains that ectopically express Str^WT^-GFP or Str^N471D^-GFP in AX2 WT and Str^−^ cells. The generation of the Str^−^ strain has been described previously (Park et al., 2013). To be able to express GFP-tagged strumpellin variants under blasticidin selection in the Str^−^ strain, we removed the blasticidin resistance cassette in this strain by transiently expressing Cre recombinase (Fig. S3A,B). All generated strains were confirmed by western blot analysis using anti-strumpellin and anti-GFP antibodies (Fig. 2A).

Strumpellin is a core member of the WASH complex, which activates the ARP2/3 complex, and also directly interacts with p97/VCP (Clemen et al., 2010; Derivery et al., 2009). Accordingly, we first analysed the expression of p97/VCP, of all WASH complex members, of the FAM21 interactor Cap32/34 and of ARPC4, as a representative of the ARP2/3 complex, in AX2, Str^−^, AX2|Str^WT^-GFP and AX2|Str^N471D^-GFP cells by quantitative reverse transcription PCR (RT-PCR). We found a strong decrease in strumpellin expression in Str^−^ cells (note that a complete knockout was confirmed at the protein level; see Fig. 2A) and confirmed its overexpression in AX2|Str^WT^-GFP and AX2|Str^N471D^-GFP cells. For all other tested genes, we observed no significant transcriptional changes (Fig. 2B). Thus, deletion or overexpression of strumpellin neither affected the expression of the other WASH complex genes nor of the tested WASH complex interactors. We next analysed the protein levels of p97/VCP, SWIP and Cap32 in AX2 and all generated strains. No changes in p97/VCP and Cap32 protein levels were detected, but we observed a significant decrease in the protein level of the core WASH complex member SWIP in Str^−^ cells (Fig. 2C,D). Re-expression of either WT or N471D strumpellin in Str^−^ cells rescued the expression of SWIP (Fig. 2C).

Next, we performed GFP-trap experiments with AX2 cells expressing GFP as a negative control, Str^−^|Str^WT^-GFP and Str^−^|Str^N471D^-GFP cells and confirmed all WASH complex proteins as interaction partners of strumpellin. All of them were among the strongest hits with the highest Student's *t*-test difference from the negative control (Table S1, green entries). No significant differences between Str^−^|Str^WT^-GFP and Str^−^|Str^N471D^-GFP cells were seen for the co-precipitated WASH complex proteins, indicating that the N471D mutation did not weaken protein-protein interactions within the WASH complex. In total, we identified 117 interaction partners for Str^WT^, among them well-known and a large number of potential new binding partners. Of the 117 identified proteins, 43 displayed either significantly reduced or no binding to Str^N471D^ (Table S1, blue entries). These constitute interesting candidates with respect to the molecular cause of SPG8.

### Strumpellin knockout cells have multiple cellular defects

**Fig. 3.**
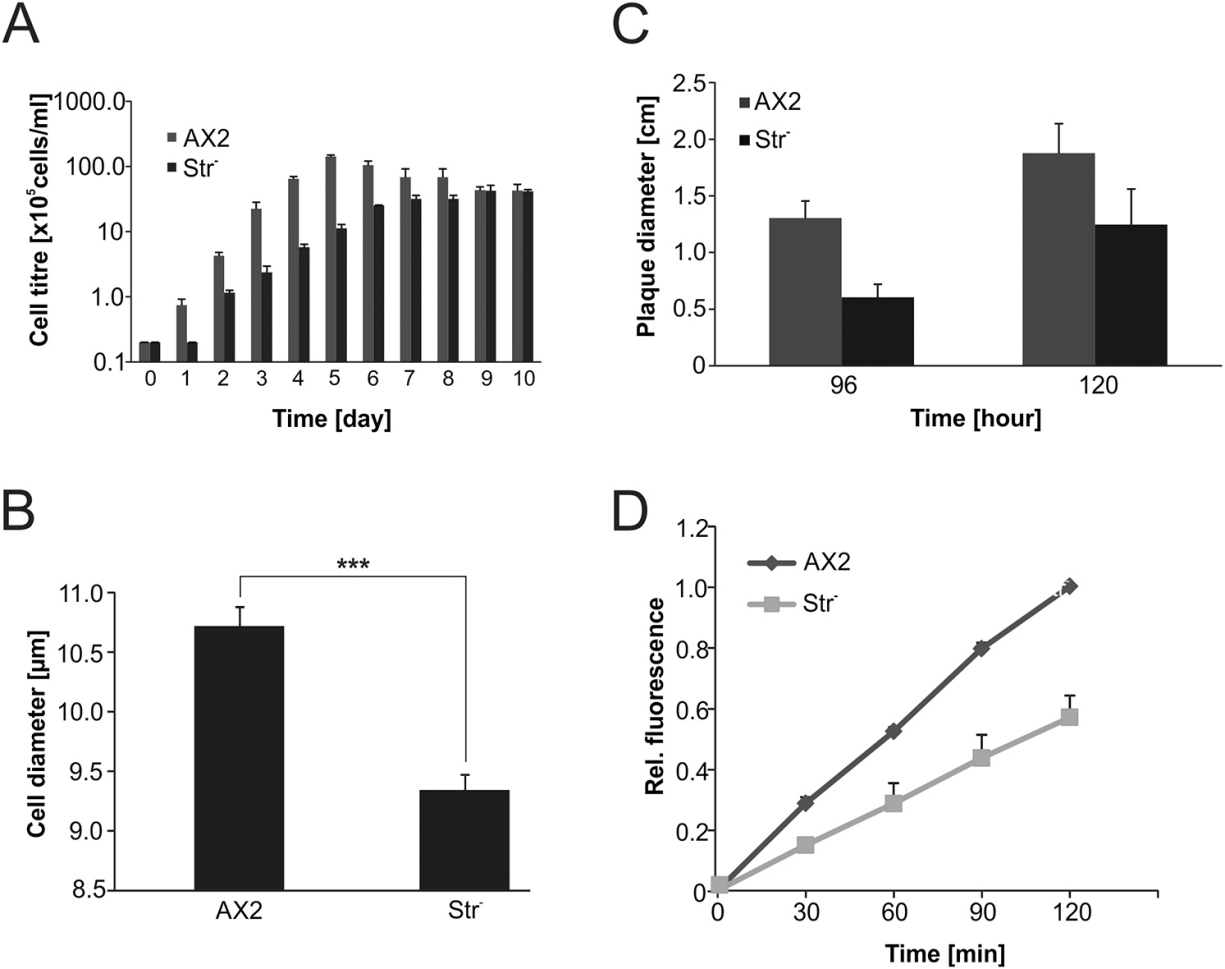
**Phenotypic analysis of Str^−^ cells.** (A) Proliferation in shaking culture. The *Str*^−^ cells displayed severe proliferation defects with a longer generation time and a lower cell density in stationary phase. Note the logarithmic scale of the *y*-axis. (B) Determination of cell size. Means±s.d. of AX2 WT and *Str*^−^ cells are depicted. For each strain, the diameters of at least 200 cells were measured. Significance was calculated using Student's *t*-test. ***P≤0.001. (C) Quantitative analysis of growth on a lawn of *K. aerogenes*. For each strain, the diameters of at least 20 plaques were determined after 96 h and 120 h of growth, respectively. Means±s.d. of three independent experiments are shown. (D) Phagocytosis assay. Quantitative analysis of the phagocytosis of TRITC-labelled yeast. Means±s.e.m. of three independent experiments are shown.

Earlier studies showed that GFP-Str colocalises with actin-coated vesicles of the endolysomal system. We noticed cytoplasmic localisation of Str^WT^-GFP and Str^N471D^-GFP and did not see a difference in localisation between WT and N471D strumpellin (Fig. S3C). Furthermore, it was shown that Str^−^ cells accumulated fluorescent dextran with the same efficiency as WT cells, but were completely blocked in exocytosis (Park et al., 2013). Because growth medium is endocytosed by macropinocytosis, i.e. in the same way as fluorescent dextran, we assayed proliferation of Str^−^ cells in shaking culture. We observed a strong proliferation defect of Str^−^ cells relative to their parent, AX2. In the logarithmic growth phase, the generation time of Str^−^ cells increased more than 40%, and the maximum cell titre was about fourfold lower than that of AX2 cells (Fig. 3A). In the process of determining cell titres in a counting chamber, we noticed that Str^−^ cells might be smaller than AX2 cells. We determined the average cell diameter of Str^−^ cells and found a ∼15% decrease in comparison to AX2 (Fig. 3B; Fig. S4A).

Recently, it was shown that the WASH complex associates with early phagosomes and is required for efficient phagocytosis (Buckley et al., 2016). We therefore analysed growth of AX2 and Str^−^ cells on a lawn of *Klebsiella aerogenes* and phagocytosis of tetramethylrhodamine (TRITC)-labelled yeast. For growth on *K. aerogenes*, we measured plaque diameters of both strains over several days and found a near twofold decrease in the plaque size of Str^−^ cells (Fig. 3C; Fig. S4B). Similarly, phagocytosis of TRITC-labelled yeast was approximately twofold less efficient in Str^−^ cells in comparison to AX2 cells (Fig. 3D).

### Overexpression of strumpellin results in cell division defects

**Fig. 4.**
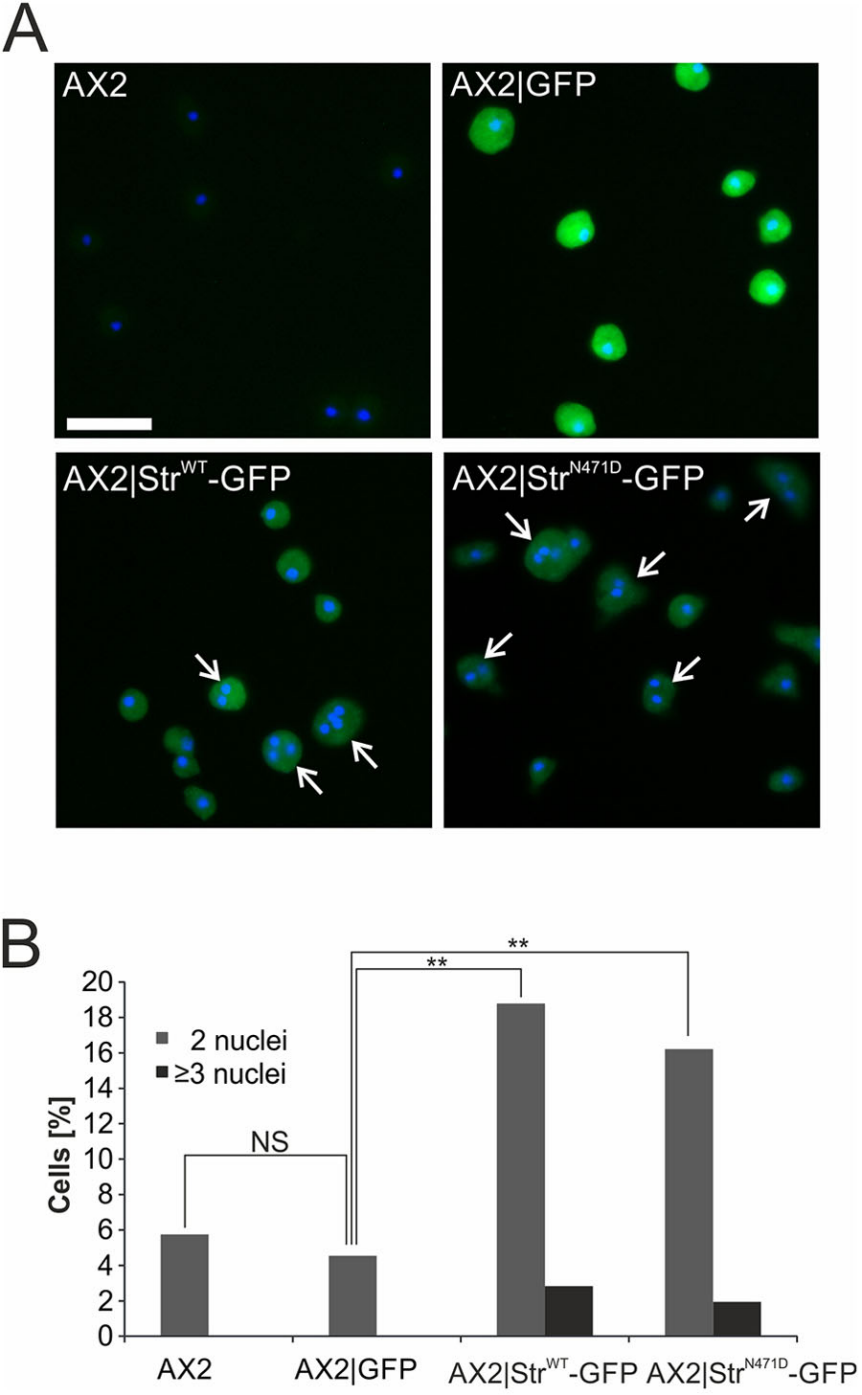
**Overexpression of strumpellin results in an increase of cells with additional nuclei.** (A) DAPI staining of nuclei in AX2, AX2|GFP, AX2|Str^WT^-GFP, AX2|Str^N471D^-GFP cells. Cells with two or more nuclei are marked by white arrows. Scale bar: 20 µm. (B) Quantification of cells with two or more nuclei. Significance was calculated using Fisher's exact test, followed by Fisher's exact test with Bonferroni correction for post hoc analyses. NS, not significant; **P≤0.01.

Fluorescence microscopy of AX2, Str^−^, AX2|Str^WT^-GFP and AX2|Str^N471D^-GFP cells suggested an increase in the number of nuclei of the latter strains. Quantification of the number of nuclei of ∼2000 cells from each strain showed that ∼5% of AX2 and AX2|GFP cells contained two nuclei and less than 0.1% had three or more nuclei. In contrast, we found for AX2|Str^WT^-GFP and AX2|Str^N471D^-GFP strains that more than 16% of the cells harboured two nuclei and ∼2% had three or more nuclei (Fig. 4A,B). This corresponds to a more than 20-fold increase in the percentage of cells with three or more nuclei in comparison to AX2 cells. The distribution of cells with one, two or more nuclei in Str^−^ cells was similar to that in AX2 cells (data not shown). Thus, overexpression of strumpellin somehow affected cell division and led to an increase in the ratio of cells with two or more nuclei. This effect was seen with both GFP-tagged constructs and was therefore independent of the strumpellin N471D mutation.

### Altered lysosome morphology in Str^−^ cells cannot be rescued by expression of Str^N471D^

**Fig. 5.**
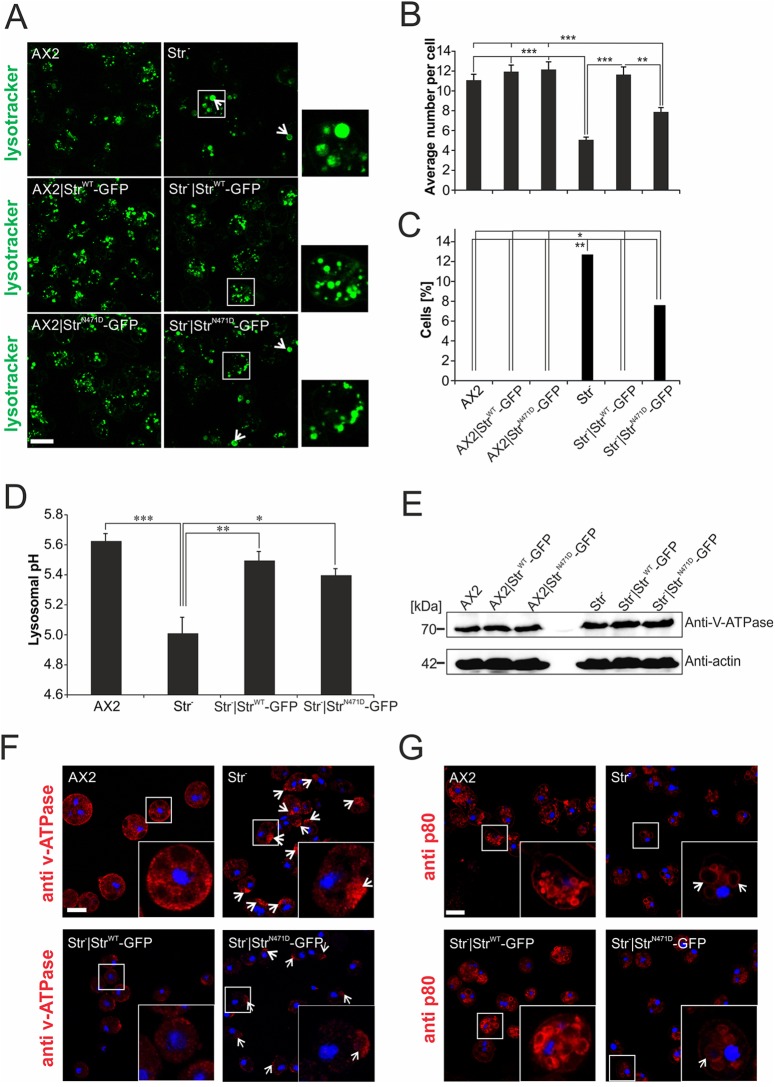
**The endolysosomal system is changed in Str^−^ and Str^N471D^-expressing cells.** (A) Lysotracker staining of live WT and mutant *D. discoideum* cells. Extraordinary large lysosomes in Str^−^ and Str^N471D^-expressing cells are marked by white arrows. (B) Average lysosome number per cell. For each strain the number of lysosomes of at least 100 cells was determined. (C) Increased number of cells with large lysosomes. The diameters of at least 100 lysosomes from each strain were determined (see also Table S2 for average diameters) and the percentage of cells containing lysosomes with a diameter exceeding 2 µm was calculated. Significance for B and C was calculated with the nonparametric Kruskal–Wallis test and the Dunn's test (with Bonferroni correction) for post hoc analyses. (D) Determination of the lysosomal pH using pH-sensitive FITC-dextran. Significance was calculated using ANOVA followed by the Tukey test. *P≤0.05; **P≤0.01; ***P≤0.001. (E) Western blot analysis of v-ATPase. Similar levels of v-ATPase were present in AX2 WT and mutant strains. (F) v-ATPase staining in fixed AX2, Str^−^, Str^−^|Str^WT^-GFP and Str^−^|Str^N471D^-GFP cells. v-ATPase-positive structures are redistributed in Str^−^ and Str^−^|Str^N471D^-GFP cells (indicated by white arrows). (G) The structure of late endosomes is changed in Str^−^ and Str^−^|Str^N471D^-GFP cells. Late endosomes were stained in fixed AX2, Str^−^, Str^−^|Str^WT^-GFP and Str^−^|Str^N471D^-GFP cells, respectively, with the anti-p80 mAb. Extraordinary large late endosomes in Str^−^ and Str^N471D^-expressing cells are marked by white arrows. See Fig. S6 for the quantitation of the mean numbers of late endosomes in the different strains. Scale bars: 10 µm.

The WASH complex is crucial for vesicle neutralisation before exocytosis (Carnell et al., 2011). We therefore quantified lysosome number and average size in AX2, AX2|Str^WT^-GFP, AX2|Str^N471D^-GFP, Str^−^, Str^−^|Str^WT^-GFP and Str^−^|Str^N471D^-GFP live cells after staining with lysotracker (Fig. 5A). Consistent with a function of the WASH complex in vesicle fission, we found that the number of lysosomes was significantly lower and their average size significantly increased in Str^−^ cells in comparison to AX2 cells. In addition, we could detect lysosomes larger than 2 µm only in a significant fraction of Str^−^ and Str^−^|Str^N471D^-GFP cells, but not in the other strains. Re-expression of Str^WT^-GFP in Str^−^ cells completely rescued the phenotype, while only a partial rescue was seen upon re-expression of Str^N471D^-GFP. Overexpression of either GFP-tagged Str^WT^ or Str^N471D^ in AX2 did not affect lysosome number and size, excluding a dominant-negative effect of the point mutation (Fig. 5B,C; Table S2). In addition, we analysed lysosomes in immortalized fibroblasts containing either the heterozygous or the homozygous N471D strumpellin knock-in or the WT strumpellin alleles. We found that the percentage of fibroblasts containing lysosomes larger than 2 µm was significantly increased in hetero- and homozygous knock-in fibroblasts (Fig. S5). This suggests that, in SPG8 patients, lysosome morphology could be affected. Next, we addressed the question whether the absence of strumpellin in the WASH complex influences vesicle neutralisation. Indeed, we found that the lysosomal pH of Str^−^ cells was significantly lower than that of AX2 cells. Re-expression of Str^WT^-GFP or Str^N471D^-GFP in Str^−^ cells led to an increase of the pH to a near normal value (Fig. 5D). Thus, the Str^N471D^ mutation apparently does not block the function of the WASH complex in vesicle neutralisation.

It was previously shown that loss of WASH leads to an arrest of lysosome maturation before the v-ATPase is removed and thus neutralisation is blocked (Carnell et al., 2011). Western blot analysis of total cellular v-ATPase did not reveal any differences between AX2, Str^−^ and both strains expressing either Str^WT^-GFP or Str^N471D^-GFP (Fig. 5E). Immunofluorescence analysis of AX2, Str^−^, Str^−^|Str^WT^-GFP and Str^−^|Str^N471D^-GFP cells stained with a monoclonal antibody (mAb) against v-ATPase subunit A revealed homogeneous labelling of cytoplasmic vesicular structures for AX2 cells. In contrast, we often observed in Str^−^ and Str^−^|Str^N471D^-GFP cells a local enrichment of the v-ATPase label (Fig. 5F). Because WASH plays an important role in late endosome maturation, we next stained AX2, Str^−^, Str^−^|Str^WT^-GFP and Str^−^|Str^N471D^-GFP cells for p80, a marker for late endosomes. We found a strong increase in size and a significant decrease in the number of late endosomes in Str^−^ cells (Fig. 5G; Fig. S6). This result suggests that fission of late endosomes is blocked in Str^−^ cells.

### Exocytosis is impaired in Str^−^ and Str^−^|Str^N471D^-GFP cells

**Fig. 6.**
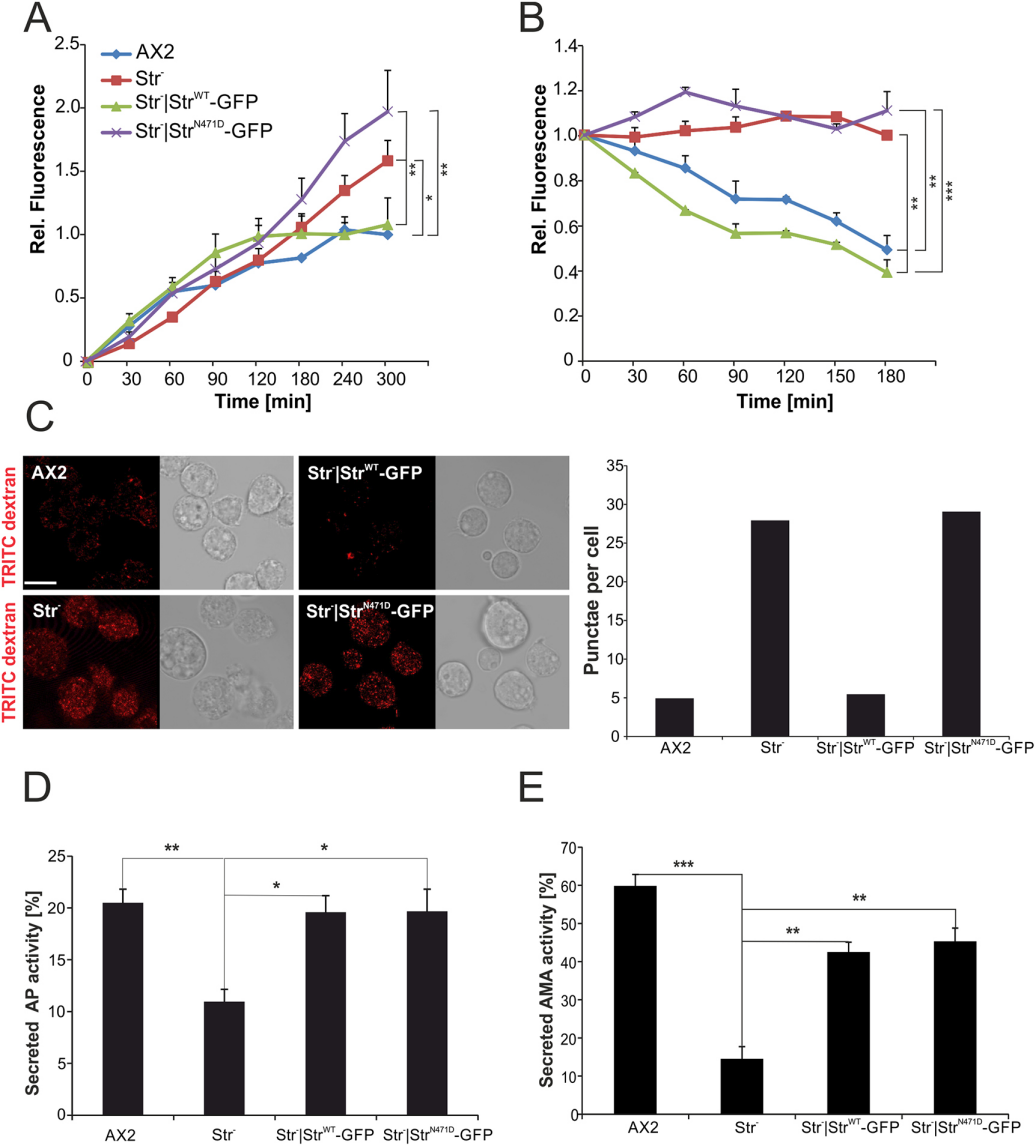
**Impaired exocytosis in Str^−^ and Str^−^|Str^N471D^-GFP cells.** (A) Pinocytosis of TRITC-labelled dextran. Relative fluorescence of AX2 WT and the respective mutant strains is shown. The fluorescence of AX2 at the 300 min time point was set to 1. Means±s.d. of three independent determinations are depicted. Significance values refer to the rise in fluorescence between 120 and 300 min. (B) Exocytosis of TRITC-labelled dextran. Relative fluorescence of AX2 WT and the respective mutant strains is shown. The fluorescence of AX2 at the 0 min time point was set to 1. Means±s.d. of three independent determinations are depicted. Significance values refer to the 180 min time point. Significance for B and C was calculated using ANOVA followed by the Tukey test. (C) Live imaging of cells after 1 h of endocytosis of TRITC-labelled dextran (left) and average number of TRITC-dextran-positive punctae (right). Mean values from at least three independent experiments are depicted. Scale bar: 10 µm. (D,E) Quantitation of acid phosphatase (AP) (D) and alpha-mannosidase (AMA) (E) activity. Intracellular and secreted AP and AMA activities, respectively, were determined and the percentage secreted activity was calculated. Means±s.e.m. of at least three independent experiments are shown. Significance was calculated using ANOVA followed by the Tukey test. *P≤0.05; **P≤0.01; ***P≤0.001.

*D. discoideum* AX2 grows efficiently in liquid medium by the macropinocytic uptake of nutrients. In contrast, Str^−^ cells have a severe growth defect, which suggests a macropinocytosis defect and/or a defect in the intracellular utilisation of nutrients. Therefore, we next investigated the endocytosis of TRITC-labelled dextran for AX2, Str^−^, Str^−^|Str^WT^-GFP, Str^−^|Str^N471D^-GFP strains. We found that, in the first 90 min, TRITC-dextran uptake was similar for all four strains and the uptake of TRITC-dextran reached a maximum after 120 min for AX2 and Str^−^|Str^WT^-GFP cells. However, Str^−^ and Str^−^|Str^N471D^-GFP cells continued to accumulate TRITC-dextran over the whole 5 h of the experiment (Fig. 6A). This result suggested that Str^−^ and Str^−^|Str^N471D^-GFP cells might have a defect in exocytosis. We therefore preloaded the cells with TRITC-dextran and then performed an exocytosis assay. The results show that, for AX2 and Str^−^|Str^WT^-GFP cells, the fluorescence was decreasing, indicating exocytosis of the indigestible TRITC-dextran. In contrast no decrease in fluorescence was seen for Str^−^ and Str^−^|Str^N471D^-GFP cells (Fig. 6B). In addition, we microscopically analysed, in parallel, the fluorescence of living cells. We observed a strong increase in fluorescence and in TRITC-dextran-positive puncta in Str^−^ and Str^−^|Str^N471D^-GFP cells, in comparison to AX2 and Str^−^|Str^WT^-GFP cells, confirming the block observed in the exocytosis assay (Fig. 6C). We next analysed the secretion of two lysosomal enzymes, acid phosphatase (AP) and alpha-mannosidase (AMA), for AX2, Str^−^, Str^−^|Str^WT^-GFP and Str^−^|Str^N471D^-GFP cells. We found, for both enzymes, a significant decrease (twofold for AP and sixfold for AMA) of the secreted activity in Str^−^ cells. For AP we observed a full, and for AMA a partial, rescue for Str^−^ cells expressing Str^WT^-GFP or Str^−^|Str^N471D^-GFP (Fig. 6D,E).

## DISCUSSION

The primary aim of our work was to analyse the cellular consequences of the Str^N471D^ mutation in the model organism *D. discoideum.* Mutant strumpellin variants are known to cause a severe and relatively pure motor form of HSP (SPG8 or Strümpell-Lorrain-Disease, OMIM #603563) (Behan and Maia, 1974; Salinas et al., 2008; Valdmanis et al., 2007). This progressive neurodegenerative disorder is clinically characterised by central motor system deficits leading to spastic paraparesis of the lower limbs (Fink, 2013). Until now, 11 point mutations and one exonic deletion in the strumpellin gene have been reported to cause SPG8 (Bogucki and Sobczyńska-Tomaszewska, 2017). Of the 11 disease-causing point mutations, eight, including the N471D mutation, which was the subject of our investigation, are localised in the central spectrin-like repeats (Fig. 1A). Spectrin repeats form three-helix bundle structures and are present in spectrin, nesprins, α-actinin and several other proteins involved in cytoskeletal structure (Liem, 2016; Noegel and Neumann, 2011). They are platforms for protein-protein or self-interactions (Djinovic-Carugo et al., 2002). Thus, a prevalent cause of SPG8 might be defects in the molecular interactions of point-mutated strumpellin within the WASH complex or with other crucial binding partners.

Previously, it was shown that *D. discoideum* knockout mutants of either CCDC53, SWIP or strumpellin have reduced amounts of WASH and FAM21 (Park et al., 2013). We found that, in the Str^−^ mutant, SWIP levels were also reduced by ∼50% (Fig. 2C,D). Similarly, RNA interference-mediated silencing of individual WASH complex members in mammalian cells, and deletion of WASH from mouse embryonic fibroblasts, resulted in a decrease of the other WASH complex proteins (Gomez et al., 2012; Jia et al., 2010). In addition, the analysis of heterozygous Str knockout mice revealed a corresponding decrease in Fam21 protein levels (Jahic et al., 2015). These results indicate that deletion of either WASH complex subunit makes the whole complex unstable and leads to degradation of its members. Our real-time PCR results revealed that the reduction in *SWIP*, *WASH* and *FAM21* in Str^−^ cells was not caused by changes in expression of these genes (Fig. 2B). Furthermore, re-expression of GFP-tagged WT or N471D strumpellin in Str^−^ cells re-established normal SWIP levels, indicating a stable WASH complex in these strains (Fig. 2C). In addition, our GFP-trap experiments revealed comparable amounts of all WASH complex proteins in the immune precipitate with Str^WT^-GFP and Str^N471D^-GFP. Stable incorporation of different point-mutated strumpellin proteins (Str^N471D^, Str^L619F^ or Str^V626F^) into the WASH complex was also observed in HeLa cells transfected with MYC-tagged WT or disease mutant strumpellin (Freeman et al., 2013). We conclude that the strumpellin N471D mutation apparently does not interfere with its binding to SWIP and possibly other WASH complex members. Thus, cellular defects in Str^−^ cells expressing Str^N471D^ were probably caused by changes in the activity of the complex or interactions of the WASH complex with other proteins.

The WASH complex as a nucleation promoting factor is crucial for the proper spatial and temporal control of actin assembly at endosomes. Surprisingly, we found that overexpression of either WT or N471D strumpellin caused an increase in multinucleated cells, suggesting problems in cell division (Fig. 4). We speculate that overexpression of strumpellin might cause ectopic actin assembly, which could lead to changes in the G- to F-actin equilibrium and to problems in cell division. An alternative possibility is that overexpression of strumpellin might affect nuclear organisation. Recently, it was found that *Drosophila* WASH is present in the nucleus and plays a key role in global nuclear organisation (Verboon et al., 2015).

Membrane trafficking in the endosomal system is an emerging pathway which is often affected in neurodegenerative diseases (Schreij et al., 2016). The WASH complex is an important regulator of vesicle trafficking and, in conjunction with the retromer, retriever and ARP2/3 complexes and actin polymerisation, is crucial for cargo sorting and delivery in distinct endosomal pathways. It is also required for the secretion of melanin but, unexpectedly, loss of strumpellin in the melanocytic lineage revealed abnormal endocytic vesicle morphology but unaffected coat colour, indicating a partially active WASH complex in the absence of strumpellin (Tyrrell et al., 2016). In mammalian cells it is essential for endosome to Golgi retrieval of the mannose 6-phosphate receptor, endosome to cell surface trafficking of e.g. transferrin, β2-adrenergic and low-density lipoprotein (LDL) receptors, and the lysosomal delivery of e.g. the EGF receptor (Bartuzi et al., 2016; Derivery et al., 2009; Duleh and Welch, 2010; Gomez and Billadeau, 2009; Gomez et al., 2012). Mis-sorting of the LDL receptor in WASH-deficient cells could cause aberrant cholesterol homeostasis. Thus, it would be worthwhile to determine plasma cholesterol levels in SPG8 patients.

**Fig. 7.**
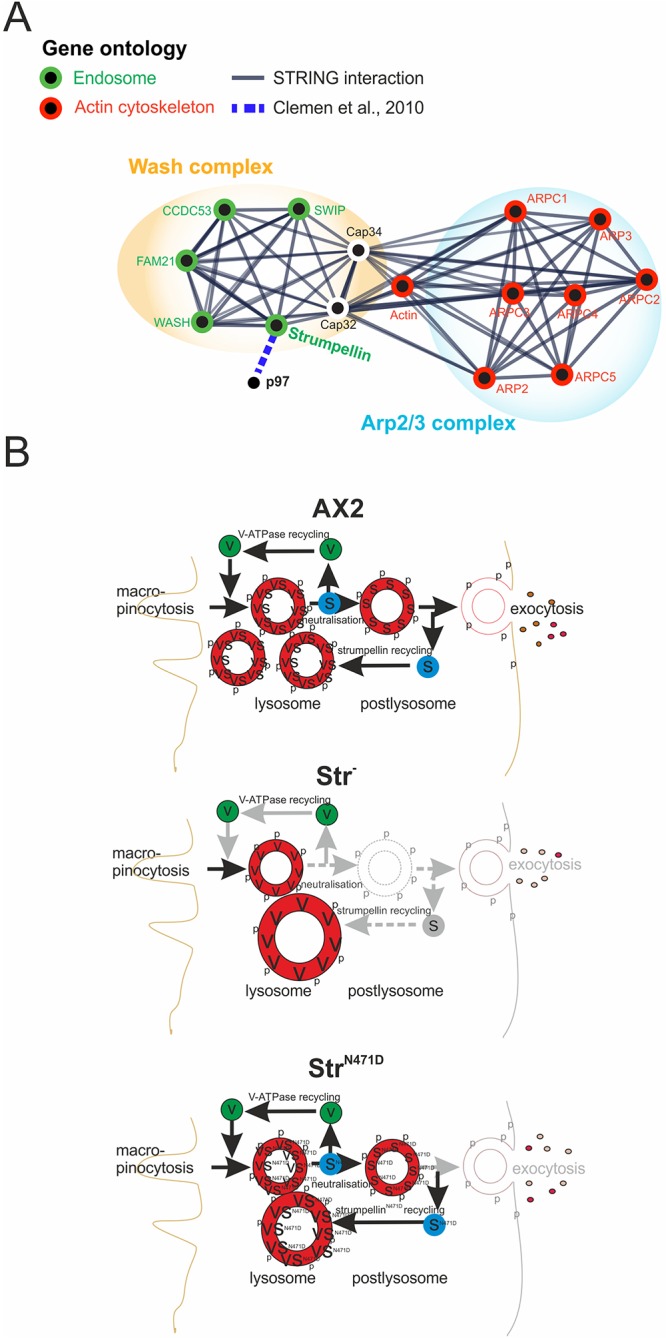
**Model for the role of strumpellin in the endocytic pathway.** The model shows the endocytic pathways in AX2 WT, Str^−^ and Str^−^|Str^N471D^ cells. Top: in AX2 cells, strumpellin is involved in v-ATPase recycling, vesicle neutralisation, exocytosis and lysosomal maturation. Middle: in contrast, in Str^−^ cells, v-ATPase retrieval, neutralisation and postlysosomal maturation are blocked. This results in fewer and larger lysosomes and causes an exocytosis defect. Bottom: expression of N471D strumpellin in Str^−^ cells rescued neutralisation but not lysosome morphology and the block in exocytosis. Black arrows denote functioning transitions; grey arrows indicate deficiencies in the respective endolysosomal transitions. p, p80; S, strumpellin; V, v-ATPase.

Sorting of proteins into more than one pathway by the WASH complex is thought to be mediated through the generation of branched actin patches, which constitute microdomains into which specific proteins are directed (Puthenveedu et al., 2010; Seaman et al., 2013). Similarly, in *D. discoideum*, the WASH complex is involved in at least two distinct endosome maturation phases. It is required in the early phase for the recycling of cell surface proteins from early macropinosomes and phagosomes and in the late phase for lysosomal recycling, v-ATPase retrieval and vesicle neutralisation before exocytosis (Buckley et al., 2016; Carnell et al., 2011; King et al., 2013). We find in Str^−^ cells, larger and fewer lysosomes and late endosomes, as evidenced by staining with lysotracker and the p80 marker for late endosomes, which were rescued by expression of Str^WT^ but not Str^N471D^ (Fig. 5A-C,G; Fig. S5). This suggests a crucial role for the Str N471 residue for the size distribution and hence dynamics of late endosomes and lysosomes. Similar morphological defects were observed in NIH3T3 cells upon silencing of WASH or the ARP2/3 complex or disruption of the actin cytoskeleton (Duleh and Welch, 2010). We also observed a decrease in lysosomal pH in Str^−^ cells. In contrast to the morphological defects, the observed decrease in lysosomal pH was rescued by expression of either WT or N471D strumpellin, indicating that the N471 residue is not important for vesicle neutralisation and v-ATPase recycling (Carnell et al., 2011) (Fig. 5D,E, Fig. 7). It was previously reported that knockout of the different WASH complex genes resulted in an exocytosis defect of undigestible material (Park et al., 2013). We confirmed this block in exocytosis in Str^−^ cells and found that it could be rescued by expression of Str^WT^ but not of Str^N471D^ (Fig. 6A-C). In contrast, a defect in the secretion of the lysosomal enzymes, AP and AMA in Str^−^ cells was rescued by expression of Str^WT^ as well as Str^N471D^ (Fig. 6D,E). We infer from these results that the N471D strumpellin mutation does not cause defects in v-ATPase recycling, vesicle neutralisation and secretion of lysosomal enzymes. However, aberrant lysosome and late endosome morphology as well as a block in exocytosis of undigestible material of Str^−^ cells could not be rescued by re-expression of Str^N471D^ (Fig. 7). Thus, depending on the endosomal pathway, strumpellin mediates interactions of the WASH complex with different binding partners. Our IP experiments in conjunction with mass-spectrometric analysis revealed 116 interacting proteins of Str^WT^-GFP. Forty-three of these did not interact with Str^N471D^-GFP (Table S1). Among these, we noted an enrichment of proteins involved in ribosome assembly and translation (seven proteins) and in ubiquitin-dependent protein degradation (five proteins). Furthermore, we identified Grp78 or Bip2 (DDB_G0276445, 78 kDa glucose-regulated protein homologue) in the immune precipitate of WT, but not N471D, strumpellin. Grp78 (also known as HSPA5) is important for endoplasmic reticulum (ER) protein folding; it was found to play a neuroprotective role in a rat model of Parkinson disease and was also identified as a new binding partner of spartin, mutations in which can cause SPG20 (Gorbatyuk et al., 2012; Milewska et al., 2009). Further interesting interacting proteins of WT, but not N471D, strumpellin were Arp1, a component of the dynactin complex, and a putative myosin light chain kinase (MLCK). Arp1 is involved in ER-to-Golgi and retrograde axonal transport, and MLCK is a cytoskeletal regulatory protein thought to be involved in myosin-mediated vesicle transport (Liu, 2017). Both proteins could provide a link to microtubule- and actin-dependent vesicle transport processes.

Neurodegenerative diseases are characterised by progressive dysfunction of neurons. Major basic processes include abnormal protein dynamics, protein aggregates and defects in endo- and/or exocytosis owing to deficiencies in the endolysosomal system. In neurons, both endo- and exocytosis are crucial for delivery of nutrients and building materials (Sambuughin et al., 2015). Because of the extremely long transport routes, proper cell homeostasis, which is mediated by a perfectly functioning endolysosomal system, is of utmost importance for motoneurons. Lysosomal dysfunction appears to be a major pathomechanism for several autosomal-recessive HSPs and additional neurodegenerative diseases (Kenney and Benarroch, 2015; Lo Giudice et al., 2014; Noreau et al., 2014; Pitcairn et al., 2018). We hypothesise that imbalances in the endolysosomal system (Fig. 7), in conjunction with disturbed protein homeostasis (Table S1), are causal for development of SPG8.

## MATERIALS AND METHODS

### *D. discoideum* strains, culture conditions and growth

*D. discoideum* AX2 was used as WT strain. The generation of strumpellin knockout cells has been described previously (Park et al., 2013). Strains expressing GFP-tagged WT and N471D strumpellin were generated by transformation of AX2 and Str^−^ cells. WT and mutant strains were grown at 21°C in liquid nutrient medium on plates (90 mm diameter), or with shaking at 160 rpm (Brink et al., 1990), or on SM agar plates with *K. aerogenes* (Williams and Newell, 1976). In case of mutant strains, the medium was supplemented with blasticidine (5 µg/ml; ICN Biomedicals, USA). Growth experiments in shaking culture and on a lawn of *K. aerogenes* were performed as described (Messling et al., 2017). Log-phase cells (2-4×10^6^ cells/ml) were used for all experiments.

### Generation of N471D strumpellin knock-in mice

We generated and used, for the purpose of this study, hetero- and homozygous N471D (c.1411_1413delAAT>insGAC) strumpellin knock-in mice B6J.129SvPas-*Stru*^tm1.1Ccrs^ and their WT siblings as well as *p53* (also known as *Trp53*) knockout mice, B6.129S2-*Trp53*^tm1Tyj^ (http://www.informatics.jax.org/allele/MGI:1857263) (Jacks et al., 1994). The N471D strumpellin knock-in mouse model was generated according to our specifications (C.S.C., R.S.) by genOway, Lyon, France. Validation of the targeted allele was performed by PCR and Southern blotting, routine genotyping was performed by PCR, and the expression of the N471D strumpellin point mutated mRNA was confirmed by RT-PCR and sequencing of the PCR products. Further details on the generation and results obtained from the analyses of the N471D strumpellin knock-in mice will be described elsewhere.

Mice were housed in isolated ventilated cages equipped with spruce granulate embedding and a nest under specific and opportunistic pathogen-free conditions at a temperature of 22±2°C, an air humidity of 50-70%, a ventilation rate of 70 air exchanges per hour and a light-dark-cycle of 12/12 h, with free access to water and food. Littermates were separated at weaning by sex and housed at a maximum of five animals per cage. Health monitoring was performed as recommended by the Federation of European Laboratory Animal Science Associations. Mice were handled in accordance with the German Animal Welfare Act (Tierschutzgesetz) as well as the German Regulation for the protection of animals used for experimental or other scientific purposes (Tierschutz-Versuchstierverordnung). All investigations were approved by the governmental office for animal care [Landesamt für Natur, Umwelt und Verbraucherschutz North Rhine-Westphalia, Recklinghausen, Germany (reference numbers 84-02.04.2014.A262 and 84-02.05.40.14.057)].

### Generation, cultivation and analysis of immortalised fibroblasts

To generate immortalised fibroblast cell lines according to Metz et al. (1995), homozygous N471D strumpellin knock-in mice were crossbred with homozygous *p53* knockout mice (Jacks et al., 1994). This resulted in offspring double-heterozygous for the N471D strumpellin knock-in and *p53* knockout alleles, which were further mated to obtain mice containing the homozygous *p53* knockout and either the heterozygous or the homozygous N471D strumpellin knock-in or the WT strumpellin alleles. The fibroblasts were obtained as a by-product from myoblast isolation according to a protocol modified from one reported previously (Rosenblatt et al., 1995; Winter et al., 2014). The immortalised fibroblast cell lines were cultivated in Dulbecco's modified Eagle medium (DMEM) supplemented with 10% fetal calf serum, 50 U/ml penicillin, 50 μg/ml streptomycin, nonessential amino acids (100 µM each), 2 mM L-glutamine, and 1 mM sodium pyruvate at 37°C and 5% CO_2_. The N471D strumpellin knock-in and p53 knockout genotypes of each fibroblast culture were verified by PCR. Cells were seeded on glass coverslips, incubated with 100 nM LysoTracker Red DND-99 (L7528, ThermoFisher Scientific, Germany) for 1 h at 37°C, washed once with 1×PBS, and images of live cells were acquired with a confocal microscope (see below). Lysosome diameters from a stack of five images were determined for more than 500 cells for each cell line in four independent experiments, and the percentage of cells with lysosomes with a diameter larger than 2 µm was determined.

### Vector construction and transformation

The pBsr-C1-GFP vector (Blau-Wasser et al., 2009) was used for the expression of full-length strumpellin and Str^N471D^ as GFP fusion proteins in *D. discoideum*. For expression of Str-GFP, the full-length *Dictyostelium* strumpellin (DDB_G0288569) complementary DNA (cDNA) was amplified by PCR, cloned into the pBsr-C1-GFP vector and the sequence verified. The strumpellin N471D point mutation was introduced with the QuikChange^®^ Site-Directed Mutagenesis Kit (Agilent Technologies, Germany), according to the instructions by the manufacturer, and confirmed by sequencing. In both fusion proteins, a linker of nine amino acids with the sequence GGSGGSGGS separated the GFP moiety from strumpellin. The plasmids were introduced into AX2 WT and Str^−^ cells by electroporation (Gaudet et al., 2007). Transformants were selected in the presence of 5 µg/ml blasticidin and cloned on *K. aerogenes* as described (Tung et al., 2010). Transformants that expressed the fusion proteins were identified by visual inspection under a fluorescence microscope followed by immunological detection of the expressed protein in western blots.

### Identification of proteins with structural homology to the strumpellin spectrin-like domain

For identification of suitable homologous models of the human strumpellin spectrin-like domain, its amino acid sequence (UniProt Q12768:248-791) was subjected to a search of known three-dimensional protein structures employing the software pGenTHREADER (Lobley et al., 2009), installed on an in-house server. The search identified Nck-associated protein in the WAVE complex (PDB 3p8c) as the template with the highest score (1e-6). Using the three-dimensional structure of Nck-associated protein 1 and the amino acid sequence of the human strumpellin spectrin-like domain, a structure-based amino acid sequence alignment was generated with SBAL (Wang et al., 2012) to appraise the structural similarity between both proteins.

### Identification of strumpellin interacting proteins

We performed co-immunoprecipitation (co-IP) experiments with AX2|GFP (negative control), Str^−^|Str^WT^-GFP and Str^−^|Str^N471D^-GFP cells. Cells (2×10^8^) of each cell line were harvested, washed twice with Soerensen phosphate buffer and the pelleted cells snap frozen with liquid nitrogen. The frozen cells were re-suspended in 2 ml lysis buffer [20 mM Tris-HCl pH 7.5, 20 mM MgCl_2_, 5% glycerol 1 mM DTT, 1× protease inhibitor cocktail (Roche, Germany) and 100 mM NaCl], followed by incubation on ice with extensive pipetting to solubilise the lysed cells. Particulate material was sedimented by centrifugation (10,000 ***g***, 10 min, 4°C). GFP-Trap beads (ChromoTek, Germany) were used to pull down GFP-tagged proteins from the supernatant according to the manufacturer's protocol. The beads were washed three times (50 mM Tris-HCl pH 7.5, 150 mM NaCl, 0.2% NP40), followed by two washes with the same buffer without NP40. On-bead digestion was performed for 30 min (50 mM HEPES pH 8.0, 2 M urea, 5 mM DTT, 1 ng/µl trypsin) followed by digestion overnight in 50 mM HEPES pH 8.0, 2 M urea, 50 mM chloracetamid, 1 ng/µl trypsin, 0.33 ng/µl LysC. Samples were acidified with 1% formic acid, purified with SDB-RP-Stage Tips, and then subjected to mass spectrometry. Four independent experiments were performed. Identified proteins with a *P*-value ≤0.05 and a Student's *t*-test difference ≥1 in the comparison between Str^WT^-GFP and GFP (negative control) were considered to represent strumpellin-interacting proteins. Those of these proteins with a *P*-value ≤0.05 in the comparison between Str^WT^-GFP and Str^N471D^-GFP, and a *P*-value ≥0.05 in the comparison between Str^N471D^-GFP and GFP, were considered as proteins that do not bind the N471D strumpellin mutant.

### Enzymatic assays of lysosomal enzymes

AMA catalyses the cleavage of the alpha form of mannose of complex sugars from glycoproteins, and AP dephosphorylates phosphate groups from phosphate esters in the lysosome. Lysosomal and secreted AMA and AP activities were determined with the colorimetric α-Mannosidase and the fluorometric Acid Phosphatase Assay Kits (Sigma-Aldrich, Germany), respectively, according to the instructions of the manufacturer. The generation of *p*-nitrophenol from the substrate *p*-nitrophenyl-α-D-mannopyranoside by AMA was determined by measuring the absorbance at 405 nm, and the generation of the fluorescent 4-methylumbelliferone from the nonfluorescent substrate, 4-methylumbelliferyl phosphate, by AP was determined by measuring the fluorescence intensity (excitation, 360 nm; emission, 440 nm) with an Infinite M1000 plate reader (Tecan, Switzerland). Three and five independent experiments, respectively, with triplicate measurements were carried out.

### Antibodies and western blotting

For the generation of specific polyclonal antibodies (pAbs) against *D. discoideum* strumpellin, the cDNA sequence encoding amino acids 30-80 was amplified and cloned into the pGEX-6P-1 bacterial expression vector. The GST fusion protein was expressed in *Escherichia coli* XL1 Blue, purified using glutathione-sepharose beads, released through cleavage with PreScission protease and used for the immunisation of rabbits. The obtained pAb str_7110 was affinity-purified (BioGenes, Germany). Protein samples from total cell lysates of 6×10^5^ cells were separated by SDS-PAGE (10, 12 or 15% polyacrylamide gel) and transferred by western blotting onto nitrocellulose membranes. Strumpellin was detected with pAb str_7110 at a 1:500 dilution, GFP with mAb K3-184-2 at a 1:50 dilution (Noegel et al., 2004), actin with mAb Act1-7 at a 1:50 dilution (Simpson et al., 1984), p97 with pAb anti-p97_8 at a 1:20,000 dilution (Arhzaouy et al., 2012), v-ATPase subunit A with mAb 221-35-2 at a 1:1 dilution (Jenne et al., 1998), cap32 with mAb188-019-31 at a 1:5 dilution (Haus et al., 1993) and SWIP with anti-SWIP pAb (animal 7762) at a 1:10,000 dilution (Arhzaouy et al., 2012). Secondary antibodies used were anti-mouse and anti-rabbit IgG conjugated with peroxidase at a 1:10,000 dilution (Sigma-Aldrich). Images were recorded and analysed using the Fluorchem SP imaging system (Alpha Innotech, USA). Background values were subtracted and the resulting intensities normalised based on actin. Three independent experiments were analysed.

### Fluorescence microscopy

Immunofluorescence microscopy of fixed cells was essentially performed as described (Tung et al., 2010). Briefly, log-phase (2-4×10^6^ cells/ml) cells were harvested, washed twice with Sorensen phosphate buffer (pH 6.0) and re-suspended at a concentration of 4×10^5^ cells/ml in the same buffer. Cells (4×10^5^) were allowed to settle down on the surface of cover slips (Ø 12 mm) inside 24-well plates for 20 min. The buffer was carefully removed, cells were fixed with pre-chilled methanol (−20°C) for 5 min, and washed three times for 5 min with PG buffer (sterile filtered PBS containing 0.1 M glycine) and twice for 15 min with PBG buffer (sterile filtered PBS containing 0.5% BSA, 0.045% fish gelatin). Fixed cells were incubated with either the p80 mAb at a 1:50 dilution (Ravanel et al., 2001) or with the ATP6V1B1 pAb (Proteintech, UK) for 1-3 h at room temperature or overnight at 4°C. Coverslips were washed five times with PBG for 5 min and one time with TBS-IF buffer (10 mM Tris-HCl, pH 7.4, 150 mM NaCl, 2 mM KCl,). Secondary antibody was either Alexa Fluor 568-conjugated goat anti-mouse IgG or goat anti-rabbit IgG at a 1:1000 dilution (ThermoFisher Scientific). Nuclei were stained with 4′,6-diamidino-2-phenylindole (DAPI, Sigma-Aldrich) at a 1:10,000 dilution. For live-cell imaging lysosomes were labelled with 0.1 µM LysoTracker^®^ Red DND-99 (ThermoFisher Scientific), a red-fluorescent dye for labelling and tracking acidic organelles in live cells. The endolysosomal system was marked with 2 mg/ml TRITC-labelled dextran as described (Rivero and Maniak, 2006). Confocal images were taken with an inverted TCS SP5 laser scanning microscope (Leica, Germany) with a 100× HCX PL APO CS1.40 oil immersion objective for fixed cells and a 63.0× HCX PL APO CS1.40 oil immersion objective for live cells. Excitation of Alexa Fluor 568, LysoTracker^®^ Red DND-99 and TRITC-labelled dextran was at 561 nm and excitation of DAPI at 405 nm. The corresponding emission wave lengths were set at 598-647 nm and 422-464 nm. Images were processed using the accompanying Leica Application Suite (LAS) software and Corel Draw X6 and quantified using ImageJ (https://imagej.nih.gov/ij/).

### Statistical analysis

All statistical analysis were performed in the R environment, version 3.3.3 (https://www.r-project.org), using the Dunn’s test (Dinno, 2015) and the lawstat package (https://cran.r-project.org/web/packages/lawstat/lawstat.pdf) (Zheng et al., 2017). For comparisons of two groups, Student's *t*-test was used. For comparisons of more than two groups, the Shapiro–Wilk and the Levene's tests were first used for the analysis of normal distribution and equality of variances. If these conditions were fulfilled, statistical analysis was performed using ANOVA followed by the Tukey test; if not, the nonparametric Kruskal–Wallis test followed by the Dunn's test (with Bonferroni correction) for post hoc analyses were used (Dinno, 2015; Spurrier, 2010). For contingency tables, Fisher's exact test was used, followed by Fisher's exact test with Bonferroni correction for post hoc analyses. Significance was indicated by *P≤0.05 (significant), **P≤0.01 (very significant) and ***P≤0.001 (extremely significant).

### Miscellaneous methods

Determination of the endolysosomal pH with fluorescein isothiocyanate (FITC)-dextran as a pH probe, and the fluorometric analysis of pinocytosis and exocytosis of TRITC-dextran at different time points were performed according to Rivero and Maniak (2006). For each experiment, triplicate measurements were taken in an Infinite M1000 plate reader (Tecan), background values were subtracted, and mean values and standard errors of, respectively, four and three independent experiments were calculated. Quantitative phagocytosis of TRITC-labelled heat-killed yeast cells was carried out as described (Xiong et al., 2015). Mean values and standard deviations of four independent experiments were calculated. RNA isolation, cDNA generation and real-time PCR (for primers, see Table S3) were performed as described (Farbrother et al., 2006).

## Supplementary Material

Supplementary information

First Person interview
