# Supplementary material for: Expression of N471D strumpellin leads to defects in the endolysosomal system
Source: Dis Model Mech. 2018 Sep 13;11(9):dmm033449. doi: 10.1242/dmm.033449 (PMC6177004; doi:10.1242/dmm.033449)
Supplement: Supplementary information [file dmm-11-033449-s1.pdf]

Disease Models & Mechanisms

FIRST PERSON

First person – Lin Song

First Person is a series of interviews with the first authors of a selection of papers published in Disease Models & Mechanisms, helping early-career researchers promote themselves alongside their papers. Lin Song is first author on 'Expression of N471D strumpellin leads to defects in the endolysosomal system', published in DMM. Lin is a guest scholar at the University of Cologne, Cologne, Germany, and worked in the lab of Prof. Dr Angelika A. Noegel and Prof. Dr Ludwig Eichinger at the University of Cologne. Her research interest is investigating translational medicine, e.g. developing new biomarkers for earlier-stage diagnoses.

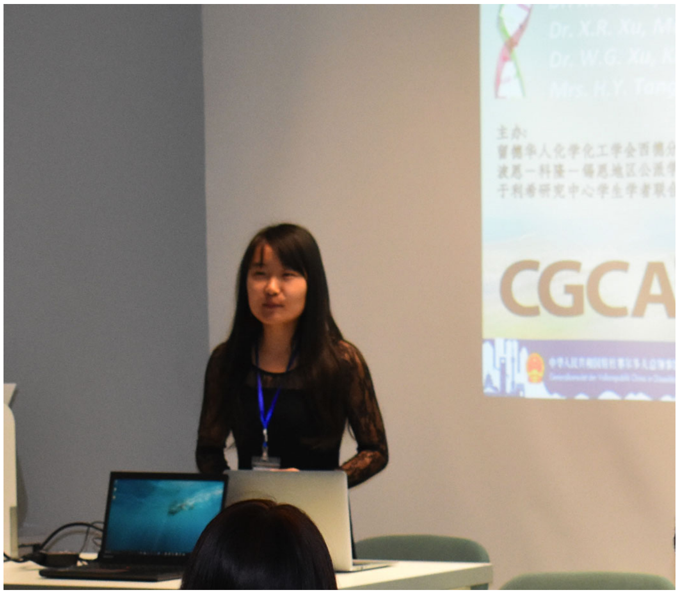

Lin Song

How would you explain the main findings of your paper to non-scientific family and friends?

We investigated the molecular mechanism of a rare genetic disease called spastic paraplegia 8. Patients suffer from severe muscle stiffness (spasticity) and, ultimately, paralysis. Despite the severe burden the disease causes for patients and relatives, little is known about the molecular cause.

Due to the rarity of available patient samples, we introduced the same mutation into *Dictyostelium* and mice, which serve as surrogates to study the disease. Remarkably, the endo-/exocytosis system in our models showed severe defects. Neurons can be extraordinarily long cells; some of them reach the length of over 1 m in humans. Therefore, neurons rely on a molecular 'conveyer belt'. This cellular trafficking system is malfunctioning and we think that it could be a cause of the disease. Restoring its molecular function could help to cure the disease and develop new therapies in humans.

What are the potential implications of these results for your field of research?

Accumulated evidence suggested that there are common pathways between different types of hereditary spastic paraplegias, and there is cross-link between different types of neurodegenerative disorders. In particular, defects in the autophagy–lysosome pathways are believed to be among the most common intracellular mechanisms underlying various neurodegenerative disorders. Investigating those key factors may, therefore, help to find novel therapy targets for spastic paraplegia 8. Currently available therapies for hereditary spastic paraplegia patients mainly consist of symptomatic medical treatment and promoting physical and emotional wellbeing.

What are the main advantages and drawbacks of the model system you have used as it relates to the disease you are investigating?

We have to admit there are certain drawbacks when we are using a haploid model (*Dictyostelium discoideum*) to investigate a very complex human disease – the physiology of *Dictyostelium* is not directly comparable to that of humans. However, the cellular properties and mechanisms are well conserved and, due to its comparatively simple handling, *Dictyostelium* allows rapid and efficient experimental investigation. More importantly, *Dictyostelium* behaves rather like a multicellular organism, which is a major benefit in contrast to *in vitro* culture of one cell type. This way we can investigate cell-cell communication in a set-up very close to the real world. Of course we don't stop there, and transfer and test our findings in the well-established mouse model.

Lin Song's contact details: University Hospital of Cologne, Medical Faculty, University of Cologne, 50931 Cologne, Germany.
E-mail: lsong2@smail.uni-koeln.de

What has surprised you the most while conducting your research?

Strumpellin is a core member of the so-called WASH complex, and a disease-related mutation in strumpellin (N471D) is involved in protein-protein interaction. So you would assume that the strumpellin^{N471D} mutation might affect other members of this complex as well – that, however, seemed not to be the case. This would suggest an entirely novel and undescribed function of strumpellin^{N471}! It will be an exciting task for the future determining what might be the 'phantom' function.

> **"[…]*Dictyostelium* behaves rather like a multicellular organism, which is a major benefit in contrast to *in vitro* culture of one cell type. This way we can investigate cell–cell communication in a set-up very close to the real world."**

Describe what you think is the most significant challenge impacting your research at this time and how will this be addressed over the next 10 years?

We consider our work in animal models only as the first step towards understanding and ultimately curing hereditary diseases – the eventual goal is always to bring results to patients. To get there, we will need to establish the role of our strumpellin mutation in

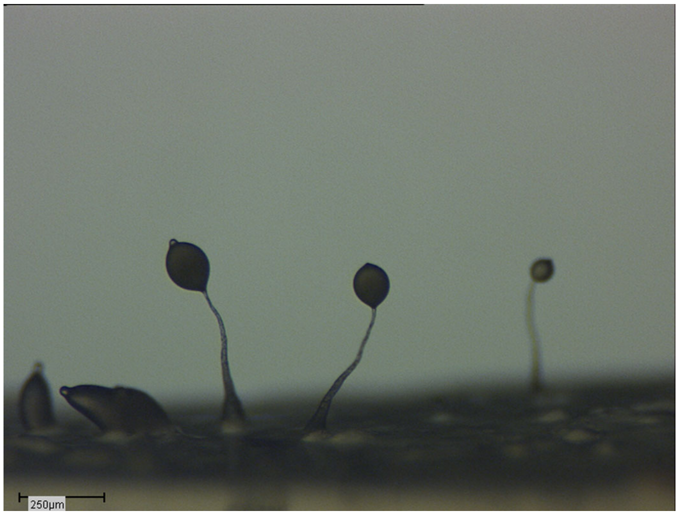

Image of *Dictyostelium discoideum*.

human tissues. At the moment we do not have these patient-derived samples available but the development of organoids is moving forward at an incredible pace! Together with the advancement of CRISPR, I am confident we can establish a corresponding organoid model system and – should the results hold up – finally move towards clinical applications.

What changes do you think could improve the professional lives of early-career scientists?

Promoting diversity and cross-discipline interactions is pivotal for an unrestricted flow of ideas – which is the fuel of fundamental research! There are already substantial efforts on the way but I believe this is a trend we need to further strengthen.

Also, free pizza and beer now and then might be a good idea as well!

What's next for you?

I finished my PhD from the University of Cologne recently and, before that, I got my Master Degree of Medicine in the field of clinical pathology and experimental oncology, and gained license work in the clinical laboratory. So, I would like to combine all my expertise to continue my research in the field of translational medicine, particularly investigating new biomarkers for the earlier stage of diagnosis. Besides my research interests, I have been actively promoting international academic exchange among scholars from different countries, and between industry and academia, for years, e.g. I regularly organize conferences, events, workshops and give lectures in different universities. In addition, I am passionate about supporting global sustainable development, e.g. I volunteered for the United Nations during my vacation. My long-term professional career plan contains these two parallel paths I mentioned above, and I have also had a dream of establishing a biomedicine company since my childhood. I am currently looking forward to joining a new astonishing team in a dynamic, international and solution-oriented environment, as well as open to all kinds of novel ideas and collaborations!

Reference

Song, L., Rijal, R., Karow, M., Stumpf, M., Hahn, O., Park, L., Insall, R., Schröder, R., Hofmann, A., Clemen, C. S. and Eichinger, L. (2018). Expression of N471D strumpellin leads to defects in the endolysosomal system. *Dis. Model. Mech.* **11**: dmm033449, doi:10.1242/dmm.033449.
